# Exosomes in Breast Cancer: Involvement in Tumor Dissemination and Prospects for Liquid Biopsy

**DOI:** 10.3390/ijms23168845

**Published:** 2022-08-09

**Authors:** Aleksei Shefer, Alena Yalovaya, Svetlana Tamkovich

**Affiliations:** 1 Institute of Chemical Biology and Fundamental Medicine, Siberian Branch of Russian Academy of Sciences, 630090 Novosibirsk, Russia; 2 V. Zelman Institute for Medicine and Psychology, Novosibirsk State University, 630090 Novosibirsk, Russia

**Keywords:** exosomes, exosomal cargo, microRNA, proteins, liquid biopsy, breast cancer

## Abstract

In women, breast cancer (BC) is the most commonly diagnosed cancer (24.5%) and the leading cause of cancer death (15.5%). Understanding how this heterogeneous disease develops and the confirm mechanisms behind tumor progression is of utmost importance. Exosomes are long-range message vesicles that mediate communication between cells in physiological conditions but also in pathology, such as breast cancer. In recent years, there has been an exponential rise in the scientific studies reporting the change in morphology and cargo of tumor-derived exosomes. Due to the transfer of biologically active molecules, such as RNA (microRNA, long non-coding RNA, mRNA, etc.) and proteins (transcription factors, enzymes, etc.) into recipient cells, these lipid bilayer 30–150 nm vesicles activate numerous signaling pathways that promote tumor development. In this review, we attempt to shed light on exosomes’ involvement in breast cancer pathogenesis (including epithelial-to-mesenchymal transition (EMT), tumor cell proliferation and motility, metastatic processes, angiogenesis stimulation, and immune system repression). Moreover, the potential use of exosomes as promising diagnostic biomarkers for liquid biopsy of breast cancer is also discussed.

## 1. Introduction

BC is the most frequently diagnosed type of cancer among women. According to the World Health Organization (WHO), every year, more than 2,261,419 cases and more than 684,996 deaths are diagnosed in the world [1]. The problem of early detection of BC consists not only in its asymptomatic development but also in the absence of reliable markers, which would facilitate early detection of the neoplastic process. Instrumental methods (mammography, method of ultrasound diagnostics, magnetic resonance imaging, etc.) have limited applicability for detection of stage I disease and BC in situ; these methods also have limitations in the discrimination of benign and malignant tumors and screening studies of healthy individuals and patients after therapy courses [2].

Mammography is one of the main methods for detecting malignant neoplasms in breasts. It is 80% to 96% reliable in detecting BC over 10 mm in diameter, but it is unable to differentiate solid from cystic neoplasms and is characterized by a 40% false-negative result rate upon detection of palpable neoplasms, which cannot detect small and solid centers of neoplasm growth. The disadvantages of mammography also include the fact that the radiation doses received do not allow for dynamic monitoring [2,3].

Ultrasonic diagnostics is the most widespread diagnostic method among women under 35. It detects BC with 92% sensitivity and 65% specificity. However, it is impossible to detect microcalcifications, which occur in 50% of BC cases, and the ultrasonic diagnosis also shows about 65% false-negative diagnoses [2].

The use of high-field MRI scans solves the problem of differentiation of benign and malignant neoplasms, allowing to differentiate them with a sensitivity of 94%, a specificity of 65%, and to assess the size and localization of any formation with a diameter over 5 mm, but they do not allow with sufficient confidence to diagnose metastatic lymph nodes, and other significant drawbacks of MRI are the high cost and lack of qualified specialists, in addition to the impossibility of its use in the presence of cardiac and vascular stents [4].

As for the latest instrumental diagnostic methods, it is worth mentioning positron emission tomography (PET). Its sensitivity in detecting BC depends on the stage of the disease and ranges from 48% to 96%, with specificity ranging from 73% to 100%. However, tumors less than 10 mm in diameter are often not detected by this method due to the limited spatial resolution of the scanners; therefore, in addition to the disadvantages of MRI, the disadvantages of PET also include the poor sensitivity of the device [5].

The main biochemical markers of malignancy nowadays are embryonic cancer antigen (CEA) and cancer antigen (CA). Increased blood CEA concentrations are observed in 30–50% of patients with BC and head and neck carcinomas, in 50–90% of patients with carcinomas of the gastrointestinal and respiratory tracts, as well as in 23% of patients with malignant tumors of connective tissue origin [6]. CA is a mucin-like glycoprotein of a 300 kDa molecular weight. This marker is used for detection and monitoring the course of BC, controlling the efficiency of therapy, and detecting recurrences. An increase in CA concentration is detected in BC in 10% of the cases at stage I, 20% at stage II, 40% at stage III, and 75% at stage IV. However, the lack of specificity, as well as the infrequent increase in concentrations at early stages, do not enable these determinants to be used for the timely detection of BC [7].

One of the prospective methods of early cancer diagnostics is the detection of tumor markers in biological fluids—liquid biopsy. Particularly for BC, the search for in-blood markers, such as mutations or epigenetic transformations in circulating DNA [8,9], circulating microRNA [10,11,12], and exosomal microRNA levels [13], and the presence of exosomal proteins [14] is ongoing. We hope that, in contrast to circulating nucleic acid analysis, comprehensive analysis of exosome proteins and microRNAs will improve the sensitivity and specificity of diagnostic systems, provide insight into the mechanisms of tumor dissemination, and enable the development of target drugs for cancer-specific therapy.

## 2. Formation and Secretion of Exosomes

**Figure 1:**
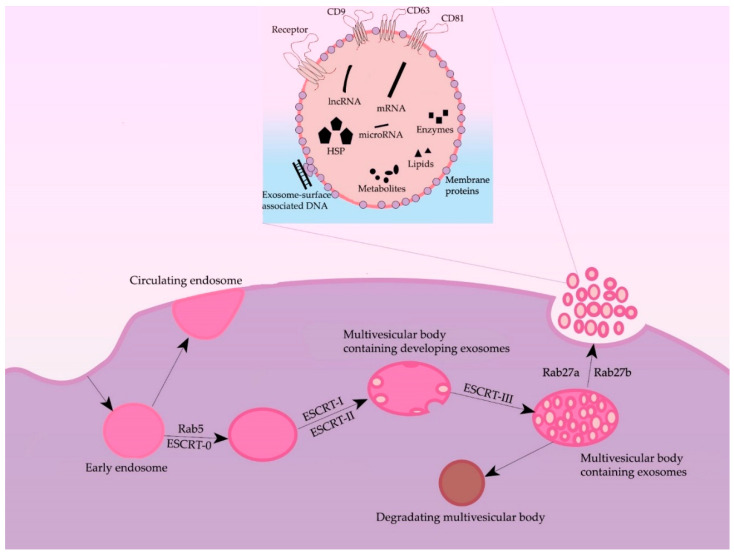
General scheme of processes of formation and secretion of exosomes and their main regulators.

Formation of exosomes—lipidic bilayer vesicles (30–150 nm) that carry CD9, CD63, and CD81 tetraspanins on their surface—begins with the invagination of plasmalemma, forming a clathrin-coated vesicle—early endosome (Figure 1). The early endosome can either be fused with the membrane back, in which case it is called a “circulating endosome”, or, after loading by nucleic acids, proteins and lipids, transforming into multiple intraluminal vesicles; intraluminal vesicles mature to the multivesicular bodies (MVBs). The main regulator of the transition from the early endosome to the multivesicular body is the small GTPase Rab5, in cooperation with the effector VPS34/p150 [15]. Once bound to Rab5, the effector proteins phosphoinositol-3-kinase, early endosomal antigen 1, and rabenzin-5 become active, which stabilizes the active form of Rab5, promoting further recognition by the FYVE domain that is part of the endosomal sorting complexes required for transport (ESCRT complex). The ESCRT-0 complex recognizes ubiquitinated proteins using the HRS and STAM1/2 heterodimer [16]. ESCRT-0 involves ESCRT-I and ESCRT-II complexes, which trigger the formation of exosomes in the membrane of the multivesicular body. Next, the ESCRT-III complex is involved, promoting detachment from the membrane and release of vesicles inside the multivesicular body [17]. The multivesicular body can then either lose its ubiquitin tag by means of a group of proteins or fuse with the lysosome and undergo degradation. Then, by the proteins Rab27A and Rab27B, the multivesicular body moves to the periphery of the cell and fuses to the membrane [18]. Moreover, the inactivation of Rab27A and Rab27B reduces exosome secretion only by half, suggesting the existence of alternative pathways for secretion. The effect of Rab5A, Rab9A, and Rab2 proteins on exosome secretion has also been shown [18] (Figure 1). Furthermore, both normal and tumor cells have been shown to increase exosome secretion under unfavorable conditions (hypoxia, heat shock, etc.) [19].

Some researchers also note an ESCRT-independent pathway of exosome formation [20]. According to some data, tetraspanins are important players in the ESCRT-independent pathway: the expression of CD9 and CD82 increased the secretion of exosomes from HEK293 cells [21]; Tspan8 tetraspanin expression did not affect the total number of secreted exosomes but changed their mRNA and protein composition [22]. CD63 is also involved in exosome biogenesis: a recent study showed that CRISPR/Cas9 knockout of CD63 led to a decrease in vesicle secretion [23]. Another protein thought to play an important role in exosome formation is the lysosome/late endosome small integral membrane protein (SIMPLE). Transfection of COS cells with this protein resulted in increased secretion of exosomes, and its mutations, in disruption of medulloblastoma formation.

The secretion of exosomes into the extracellular space is caused by the fusion of the MVBs with the plasmalemma. The fusion process begins with the synaptotagmin protein interacting with calcium receptors, causing the multivesicular body binding to the trans-SNARE complex, followed by exosomes secretion into the extracellular environment [24].

**Figure 2:**
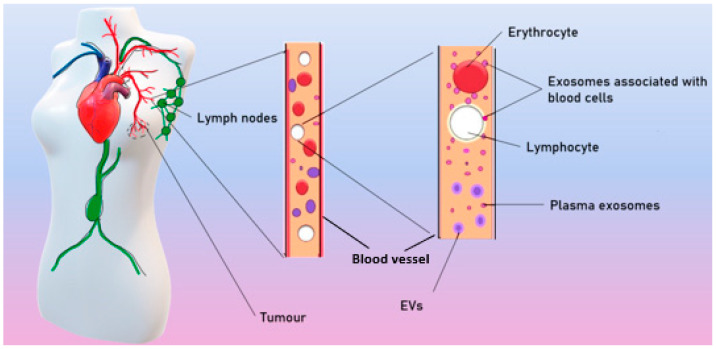
Spreading of tumor exosomes in organism.

Exosomes are ordinarily released by different cell types and can both locally affect nearby cells or distally affect cells, spreading to the tissues with the blood and lymph. As a result, exosomes are found in all biological fluids of healthy individuals [25]. In patients with BC, tumor exosomes spread in the organism and are found in the blood [26], ascites [27], tears [28], and breast milk [29] (Figure 2)**.**

According to different published sources, it is known that there are from 50 to 300 million exosomes per 1 mL of blood in healthy donors [26]. Theoretically, each exosome contains lipids, no more than 100 proteins, and no more than 1000 microRNA [30]. Moreover, a number of studies have shown that exosomes in the blood circulate in a free form but are also associated with surface of blood cells (csbExos) [13,26], allowing them to move through the organism over long distances. It has also been shown that csbExos contain twice as much RNA as exosomes circulating in blood plasma [13], which may indirectly indicate a significant role of exosomes associated with blood cells in tumor progression.

Several mechanisms have been suggested for transfer of exosomal cargo to recipient cells. There are (i) fusion of exosomes with the cellular membrane, leading to the release of exosomal content into the cytoplasm of the recipient cell; (ii) juxtracrine signaling through ligand–receptor interactions; (iii) endocytosis by phagocytosis [31].

## 3. Morphology and Content of Exosomes

**Figure 3:**
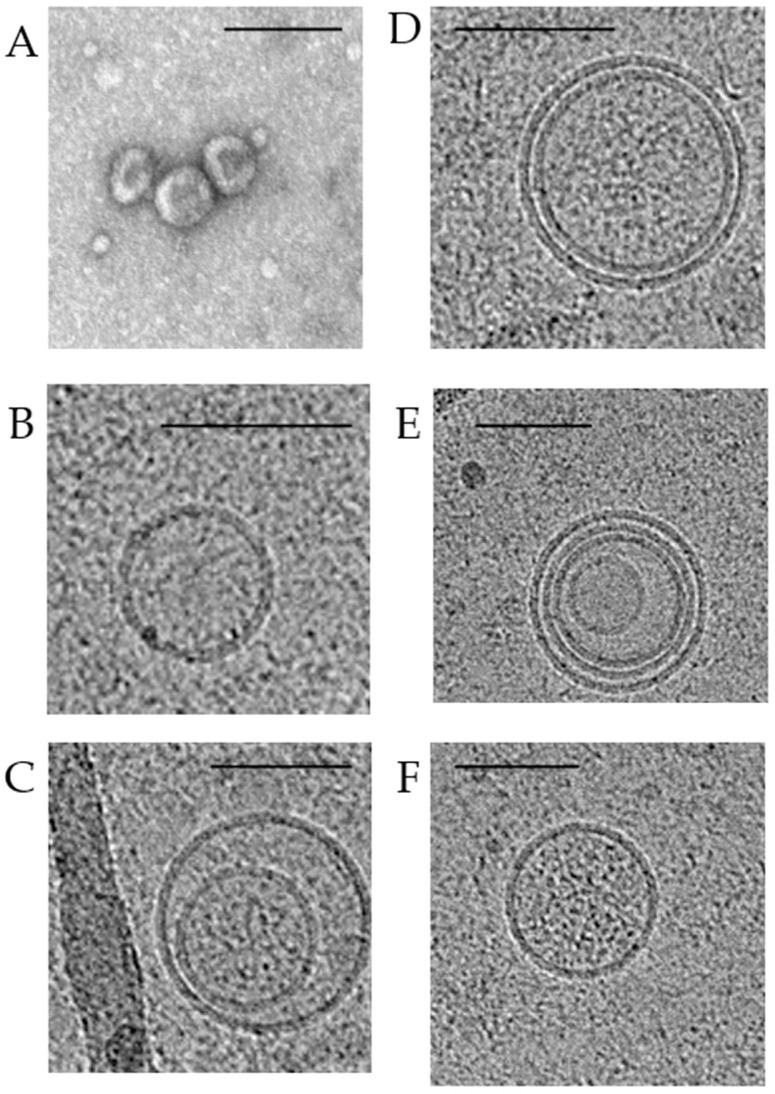
Transmission and cryo-electron microscopy images of exosomes isolated from pooled samples of BC patients’ plasma. Typical transmission electron microscopy view of exosomes (**A**). Cryo-electron microscopy images of exosomes: single vesicles (**B**); double vesicles (**C**); double-membrane vesicles (**D**); multilayer vesicles (**E**); vesicles with electron dense cargo in lumen (**F**). Scale bars are 100 nm.

Using transmission electron microscopy, it has long been known that exosomes are cup-shaped and their morphology does not differ under physiological and pathological conditions (Figure 3A). In recent years, cryo-electron microscopy has established a wide range of exosome morphology with lipid bilayers and vesicular internal structures [32,33]. Moreover, the ratio of such exosomes in BC has been shown to change both in plasma and in total blood (the samples contained plasma exosomes and csbExos). In particular, in plasma of oncological patients, compared with healthy women, the proportion of single vesicles (Figure 3B) decreases (from 91% to 81%) and the content of double exosomes (Figure 3C) increases (from 3% to 8%), as well as double membrane exosomes (Figure 3D) (from 0% to 3%). More significant changes in the representation of various structures of exosomes were found in the total blood: in BC, compared with the norm, the proportion of single vesicles increases (from 37% to 70%), while the proportion of double exosomes decreases (from 20% to 10%), as well as exosomes with double (from 22% to 9%) or multilayer membranes (Figure 3E) (from 8% to 5%), and with electron dense cargo (Figure 3F) (from 13% to 6%) [34].

Since total blood exosomes contain two fractions (cell-free exosomes and blood csbExo), the revealed phenomenon indicates that exosomes of various morphologies predominate on the surface of blood cells. Apparently, there are subpopulations of exosomes with different functions, and blood cells act as their carriers to distant targets. In BC, not only does the content of exosomes change due to the specific sorting of biologically active molecules but there is also a redistribution of various subpopulations of exosomes in the blood between the plasma and the surface of blood cells [14].

The composition of RNA and exosome proteins is being actively studied, but, at the moment, there is quite little data on the sorting of these molecules into vesicles. Exosomes are enriched with some microRNAs compared to mother cells, which suggests that microRNAs can be specifically sorted into exosomes. In particular, a sequence motif was identified that controls the transport of RNA to future exosomes through binding to the A2B1 protein heterogeneous nuclear nucleoprotein (hnRNPA2B1), while the exosomal hnRNPA2B1 is sumoylated (post-transcriptional ubiquitin-like modification by proteins of the SUMO family, SUMOylation) [35]. Apparently, this modification by small ubiquitin-like proteins is necessary for its microRNA binding. It has also been shown that the KRAS GTPase plays a role in the sorting of microRNAs into exosomes. Exosomes of the colorectal cancer cell line with mutant *KRAS* (DKO-1, DLD-1) showed a microRNA expression profile significantly different from exosomes of cells with wild-type *KRAS* (DKs-8) [36]. There is reason to believe that some mRNAs are also selectively transported to exosomes. Thus, exosomal mRNAs are enriched with 3′-untranslated regions, and it is assumed that this may play a role in sorting mRNAs into vesicles. Indeed, it has been shown that exosomes are enriched with mRNA and long non-coding RNAs containing ACCAGCCU, CAGUGAGC, and UAAUCCCA motifs, which are selectively transported to exosomes due to specific binding to YB-1 and NSUN2 proteins [37]. Since the composition of exosomes shows an increased content of ubiquitinylated proteins, there is also an opinion that ubiquitination may be a marker of the selection of proteins into exosomes.

### 3.1. Metabolome of Exosomes

The metabolome of exosomes represents the intermediate of the final point of processes in parent cells, so it shows the phenotype of the organism. It is known that metabolome of blood-derived exosomes contains nucleotides and nucleosides, peptide conjugates, fatty, amino, and carboxylic acids, phenols, steroids, less abundant sugars, and alcohols [38,39,40,41]. Moreover, it has been shown that exosomes secreted by tumor cells contain elevated levels of pyruvate and lactate, while α-ketoglutarate, malate, glutamate, and fumarate are decreased [42,43].

One of the recent studies has shown a significant difference in exosomal metabolome between pseudo-normal epitheliocyte cell line MCF-10A and BC cell line MCF-7. It has been shown that, in MCF-7-cells-derived exosomes, 42 metabolites were downregulated and 43 metabolites were upregulated compared to MCF-10A-derived exosomes. It was particularly shown that glutamine and aspartic acid are significantly downregulated in BC cells, which can be used for distinguishing BC patients [44,45].

### 3.2. Lipidome of Exosomes

The lipid composition of exosomes is very similar to those in secreting cells, especially to the plasma membrane. The most commonly found lipids in exosomes are sphingomyelin, phospholipids, ganglioside GM3, and cholesterol, but the relative abundance can differ due to the secreting cell type and stage or the function of exosome [46,47,48,49]. It is known that lipids on the exosome membrane are distributed asymmetrically; therefore, sphingomyelin is commonly found on the external side of the membrane, while phosphatidylserine is commonly located on the interior side [50]. However, it has been shown that exosomes secreted by tumor cells demonstrate the presence of phosphatidylserine on the external side of the membrane [51]. Furthermore, it was shown that several exosomal lipids can mediate signal transduction; for example, diglycerol is a second messenger in the protein kinase C signal pathway, which is involved in tumor angiogenesis [52]; such activity has been shown for highly metastatic triple-negative BC cell line D3H2LN [53]. It was also shown that the content of sphingomyelin is higher, while the content of phosphatidylserine is lower in the BC-cells-derived exosomes in comparison with normal epithelycyte-derived exosomes [53].

### 3.3. Proteome of Exosomes

With the growing number of publications, it has become common to divide exosome proteins into nonspecific proteins (present in most exosomes regardless of the source of origin), such as membrane tetraspanins, CD63, CD9, and CD81 proteins, responsible for transport and binding to the target cell (syntenin-1, GTPases, annexin), adhesion proteins (integrins), heat shock proteins (Hsp 70, Hsp 90), proteins involved in multivesicular body biogenesis (Alix, TSG-101), and tissue-specific proteins—CD37 and CD53 for leukocytes, AMPK α1 for adipocytes, etc. [54]. It should be noted that all proteins found in exosomes are present in the cytoplasm, in endocytosis vesicles, and on the cytoplasmic membrane. In general, when describing the protein composition of exosomes, one cannot fail to note the high heterogeneity of the composition of these vesicles depending on the conditions in which the secreting cells are located (including stress conditions, such as heat shock, genotoxic stress, oxidative stress, hypoxia), the type of secreting cells and secreted cells, vesicles, the sites of secretion, and the biological fluid in which they circulate, as well as the methods of isolation and purification used [55,56]. Based on the ExoCarta database (www.exocarta.org, accessed on 10 January 2022), which contains data from independent studies of proteins, lipids, microRNAs, and mRNAs contained in exosomes as of the beginning of 2022, 20 proteins of most specific exosomes can be identified (Table 1).

In spite of the high heterogeneity of exosome proteins in both normal and pathological conditions, it is possible to identify proteins characteristic of only one group. In particular, out of the 223 proteins identified in blood exosomes, only 34 (15%) proteins were universal for healthy women and BC patients [26].

Research on the identification of tumor-specific exosome proteins was also performed on cell cultures. In one of the works, the proteome of exosomes of pseudo-normal MCF-10A cell line and MDA-MB-231 line of triple-negative BC subtype were compared. In this work, 986 proteins were identified, of which 726 appeared unique to the transformed cell line, 260 appeared universal, and 121 proteins appeared unique to the pseudo-normal epitheliocyte line [57].

### 3.4. Nucleic Acids Transported by Exosomes

It has been shown that, in addition to proteins, exosomes carry DNA and various types of RNA: tRNA, rRNA, snRNA, and others, of which microRNAs and long non-coding RNAs are currently attracting the most attention.

According to the Exocarta database (www.exocarta.org, accessed on 10 January 2022), exosomes are involved in the transport of more than 2838 microRNAs and 3408 mRNAs, the composition of which in microvesicles depends on the composition of parental cells and the general state of the organism. The transfer of intact mRNAs by exosomes from donor to recipient cells can participate in the exchange of phenotypic traits between cells as recipient cells receive mRNAs that are not initially expressed in them [12].

Long non-coding RNAs (lncRNAs) also play a significant role in tumor development: they regulate transcription by binding to enhancer regions. In addition, lncRNAs are involved in chromatin modification, mRNA splicing, and maintaining protein stability by regulating cell differentiation, gene imprinting, and the antiviral response [58].

Using 4T1 cell cultures, Zhang et al. have shown that MALAT 1 IncRNA (metastasis-associated lung adenocarcinoma transcript 1) was shown to be overrepresented in exosomes secreted by breast carcinoma cells. Further in vivo experiments have shown that, inside target cells, MALAT1 caused increased cell proliferation, promoting tumor growth [59].

It has also been shown that lncRNAs can act as blockers of microRNA repressor activity by binding target transcripts to the 3′UTR. An increased level of lncRNA in tumor cells increases their resistance to tamoxifen (lncRNA MALAT1 and CCAT2; miR-221, miR-222, miR,26a, miR-P29a, miR-29b) and trastuzumab (lncRNA, miR-16, and miR-155). Moreover, lncRNAs are involved in the binding of the HOTAIR gene to the histone-modifying complexes PRC2 and LSD1 to promote H3K27 histone methylation and H3K4 demethylation, which lead to the shutdown of target genes and promote BC metastasis [58].

According to some data, exosomes can transfer DNA: genomic dsDNA fragments [60], ssDNA fragments [61], and fragmented mitochondrial DNA [62]. It has been shown that dsDNA is the majority fraction of DNA from cancer-cell-line-derived exosomes (>50%) and reflects the mutational status of parental tumor cells [63]. However, many researchers suppose that the presence of DNA inside exosomes is an artifact related to the contamination of vesicle preparations by apoptotic blebs of similar size. Recent works demonstrate the presence of DNA on the exosomal outlier membrane surface, which binds to the vesicle membrane through DNA-binding proteins [64,65]. Exosomes of infected cells also often carry pathogen DNA, inducing an adaptive immune response in lymphocytes by triggering the cGAS-STING signaling pathway and similarly triggering the anti-tumor immune response [66,67].

## 4. Role of Exosomes in Breast Tumor Progression

Although malignant cells themselves are the major source of tumorigenesis, their interactions with the tumor microenvironment are critical for the progression from a single tumor mass to distant metastases. Derived from cancer cells, exosomes mediate EMT, malignant cell proliferation and motility, metastatic processes, angiogenesis stimulation, and immune system repression.

### 4.1. Epithelial–Mesenchymal Transition

EMT is a necessary precondition for metastasis; it is usually followed by the transition of epithelial to mesenchymal cells. Such a process is crucial for the metastasis process so that epithelial cells could acquire the ability to move through blood vessels [68]. The EMT program is usually activated during metastasis and tumor invasion; hence, the genetic changes and molecular mechanisms by which cancer cells acquire invasiveness and their subsequent metastatic ability have been areas of intensive research.

Tumor-derived exosomes also carry some oncogenic microRNAs, such as miR-23a, miR-5100, miR-19b-3p, and miR-21, which control the EMT process [69]. The levels of expression of such microRNAs in blood could be the basis for various exosomes-based diagnostics [69]. For example, discussed above as metastatic, causing proliferation, and increasing migration, exosome-encapsulated miR-1910-3 p is a biomarker of BC that circulates in the blood [70].

MiR-34a, which is a p53 transcription target, is reported to inhibit the aggressiveness of BC cells by inhibiting EMT and zinc finger transcription inhibitor Snail [71]. The balance between the expression levels of the miR-22 and miR-200 families also regulates the EMT phenotype in BC, so these microRNAs are considered an important factor in controlling the first steps of metastasis [72,73].

Cancer-associated fibroblast (CAF)-derived exosomes carrying miR-181d-5p are suggested to enhance proliferation, invasion, migration, and EMT and suppress apoptosis of BC cells through regulation of *CDX2* and *HOXA5* genes. It was announced that overexpression of HOXA5 inhibits proliferation, migration, invasion, and EMT and induces apoptosis of MCF-7 cells. Transcription factor CDX2 binds to the Hoxa5 promoter and promotes HOXA5 expression, while miR-181d-5p mimics CDX2 to reduce expression [74].

The EMT phenotype can also be induced by miR-103/107 in BC cells. It was found that miR-103/107 weakened microRNA biosynthesis by targeting the gene encoding Dicer, leading to the total downregulation of microRNAs, including the miR-200 family, as well as the further development of EMT and the metastatic phenotype of epithelial tumor cells [75].

### 4.2. Proliferation

Reaching the Hayflick limit, most of the cells enter a process of programmed self-liquidation, called apoptosis. Malignant tumor cells are able to avoid apoptosis by increasing the expression level of many antiapoptotic genes of the *BCL2* family, as well as by inducing oncogenic mutations in *p53* and hyperexpression of apoptosis inhibitor proteins. As a result, BC cells go through the stages of the cell cycle faster and the level of cell proliferation increases [76]. Cyclins and cyclin-dependent kinases have also been shown to have a regulatory role in accelerating the cell cycle as the expression level of these kinases affects changes in the cell cycle [77].

The results of in vitro uptake of exosomes derived from murine BC cell line 4T1 showed equal efficiency for CD133+ and CD133- cell lines [78]. CD133+ is a marker of cancer stem cells, and its expression is associated with a poor prognosis and chemoresistance in some types of solid tumors, including BC. Meanwhile, in vitro analysis of cell proliferation showed that exosomes from 4T1 cells significantly increased proliferation of CD133+ cells but had no effect on CD133- cells. In addition, the level of apoptosis in CD133-positive cells after treatment by doxorubicin (an apoptosis-inducing drug) was significantly suppressed by exosomes originating from 4T1, whereas, in CD133-negative cells, it did not occur. The results allowed the authors to suggest that exosomes from tumor cells may function as pro-tumor factors, contributing to active proliferation and suppression of apoptosis of CD133+ tumor stem cells [78].

One of the recent research studies has shown that exosomal miR-500a-5p derived by cancer-associated fibroblast significantly promotes proliferation in MDA-MB-231 and MCF-7 BC cell lines via targeting the USP28 protein [79]. Furthermore, the level of MiR-1910-3p is significantly higher in BC cell lines MDA-MB-231 and MCF-7 compared to pseudonormal epitheliocytes line MCF-10A; furthermore, it was shown that miR-1910-3p promotes cell proliferation in vivo by activating the NF-kB signal pathway [68].

The study of blood exosomes revealed that the addition of total blood exosomes (from the plasma and from blood cell surface) from BC patients led to a statistically significant increase in the mitotic event number of MCF-10A cells in contrast to plasma exosomes. These results indirectly indicate that csbExos are responsible for the stimulation of cell proliferation. It was an unexpected finding that total blood exosomes of healthy females stimulated the proliferation of MCF10A too [34]. Since most exosomes in the blood of cancer patients are of non-tumor origin, it is likely that, without attracting attention to them, exosomes from normal cells play a significant role in tumor dissemination.

### 4.3. Cell Motility

In addition to the effect on cell proliferation, tumor-derived exosomes can also change the ability of recipient cells to migrate. For example, miR-9, delivered by exosomes from cancer cells to fibroblasts, leads to their transformation into tumor-associated fibroblasts with increased cellular motility [80].

Further, miR-135a has been reported to be highly expressed in metastatic breast tumors. It was found that the expression of miR-135a is necessary for the migration and invasion of BC cells, but not for their proliferation. *HOXA10* encodes a transcription factor necessary for embryonic development and is a metastatic inhibitor in BC. It has been shown to be a direct target of miR-135a in BC cells. The study has truly demonstrated that miR-135a inhibits HOXA10 expression at both mRNA and protein levels, and its ability to promote cell migration and invasion is partially reversed by HOXA10 overexpression [81].

Further, miR-130 delivered into macrophages by exosomal transport has been reposted to cause M1 polarization (macrophage repolarization from M1 to M2 phenotype). It was also shown that the phagocytosis ability of macrophages enhanced after being treated by microRNA-loaded exosomes. Thus, migration and invasion assays demonstrated reduced ability of 4T1 BC cells for migration and invasion after macrophages reprogramming [82].

It is shown that miR-148a and miR-16 upregulate MDA-231 breast cancer cells’ motility via targeting *CCNE1*, *Twist1*, and *Wnt10b* [83]. Furthermore, MiR-7641 is upregulated in highly metastatic BC cell line MDA-MB-231 in comparison to non-metastatic MCF-7 cells and is shown to upregulate cell motility [84].

The study of the effect of blood exosomes on cell motility showed that the addition of exosomes from the total blood of healthy females or from the plasma and total blood of BC patients resulted in a significant increase in the motile MCF-10A cell number compared to the negative control. MicroRNA analysis associated with cell motility revealed the increased level of miR-92a in plasma exosomes and level miR-25-3p in total blood exosomes of BC patients compared to healthy females [34].

Adipocyte-derived exosomes from cell line 3T-L1A were shown to increase BC cell motility in cell lines MCF-7 and MDA-MB-231 via increasing the level of HIV-1α [85].

### 4.4. Metastasis

The mortality rate from BC is determined by the development of distant metastases to the lung, brain, bones, and liver. Predicting the site of probable metastasis is important for determining the therapeutic algorithm that could prevent the spread of tumor cells. The capacity of exosomes to affect the invasiveness and, consequently, metastasis of cancer tumors has been shown both in vitro and in vivo [86]. Moreover, exosome cargo impacts tumor niche establishment and regulates the tropism of metastasis [87].

Analysis of MCF-7 cell exosome proteins (characterized by low metastatic potential) showed increased content of tetraspanin superfamily proteins (Tetraspanin-14, CD9, CD63, and CD81, which enhance cell adhesion and decrease the propensity for migration and metastasis, and, in exosomes of MDA-MB-231 line (characterized by high metastatic potential), proteins enhancing cell motility (Vimentin, Galectin-3-binding protein, Annexin A1, Plectin, Protein CYR61, EGF-like repeat, and discolding I-like domain containing protein, Filamin-D, Protein-glutamine gamma-glutamyltransferase 2) were hyperrepresented [88]. Exosomes have also been shown to carry survivin, enhancing the expression of SOD1, which controls differentiation of fibroblastic cells to myofibroblasts, disrupting fibroblast adhesion, allowing metastasizing tumor cells to invade new sites, and increasing proliferation of BC cells [89]. Exosomal aspartate-β-hydroxylase is also involved in BC metastasis: it has been shown that the enzyme triggers the Notch signaling pathway that induces exosome secretion and increases cell aggressiveness.

Molecular insight into exosome cargo has disclosed the pivotal roles of microRNAs in BC metastasis; for example, it has been widely demonstrated that exosomes secreted by BC cells promote CAFs activation by the miR-146a/*TXNIP* axis to start the *Wnt* pathway, which, in turn, increases the metastasis and invasiveness of BC cells [90]. Further, miRNA-9, carried by exosomes, is shown to be a pro-metastatic microRNA and is abundant in several BC cell lines. Additionally, it is able to induce switches in the cancer phenotype in normal fibroblasts and thus promotes tumorigenesis. However, some studies suggest that miR-9 also acts as anti-oncogene in BC proliferation; that is, it inhibits the occurrence of BC at an early stage of the disease [91]. For instance, miR-10 was first determined as a major regulator of metastasis in BC. The investigations showed that the expression levels of miR-10b were much higher in metastatic than non-metastatic BC cell lines. Ectopic expression of miR-10b in non-metastatic BC cell lines induces upregulation of Ras homologous gene family members by direct targeting of the gene encoding homeobox D10, which leads to the promotion of invasiveness and metastasis [91].

Further, miR-19a, which belongs to the miR-17-92 cluster, is reported to have a tumor-promoting role in multiple types of cancers. It is also known to target PTEN and is predicted to target ER. MiR-19a expression is reported to positively correlate with osteolytic bone metastasis in vivo, strongly suggesting a role of miR-19a as a key modulator of tumor microenvironment in the process of bone metastasis of BC. Therefore, exosomal miR-19a mediates cell–cell communication between BC cells, promoting the vicious cycle of bone metastasis in ER + BC [92].

Further, miR-21 and miR-200 have been shown to be differentially expressed in BC cells’ exosomes. Some studies performed using exosomes isolated from tear liquid have suggested that the amounts of miR-21, and miR-200c were significantly higher in tear exosomes isolated from patients with metastatic BC than those of healthy controls. Those data assure tears as an alternative biological liquid that requires less purification [28].

BC cells produce exosomes carrying miR-122 that is shown to stimulate metastasis by forming the pre-metastatic niche. In this case, these exosomes block glucose uptake by pre-metastatic niche cells and breaking the energy metabolism, promoting cancer cells to progress [93].

Further, miR-1910-3p contained in exosomes is shown to activate proliferation, migration, and autophagy. Meanwhile, it was reported to inhibit apoptosis in TNBC and ER- and PR-positive BC cells. Studies suggested that miR-1910-3p, carried by TNBC and ER- and PR-positive BC cells inhibits MTMR3, activating the NF-κB signaling pathway [68].

Furthermore, it was shown that breast cancer cells of MDA-MB-231 treated by exosomes from the 3T-L1A cell line show increased metastatic potential during in vivo experiments on mice [85].

### 4.5. Angiogenesis

Exosomes secreted by tumor cells are involved in active angiogenesis, caused by hypoxia. It was shown that BC cell lines MCF-7 and MDA-MB-231 show increased levels of secreted exosomes in a 0.1% O_2_ environment compared to a 1% O_2_ environment [94]. Particularly, exosomes secreted by BC cells stimulate transformation of mesenchymal stem cells to myofibroblasts through the SMAD-mediated pathway (transforming growth factor signaling pathway). Myofibroblasts are among the core cells in the tumor, being involved in the processes of vascular reorganization [95]. Furthermore, exosomes secreted by BC tumor cells are known to induce fibroblast activation, which stimulates [96]. Assessment of the angiogenic potential of total blood exosomes and plasma exosomes of BC patients showed a comparable effect: they stimulated the formation of capillary-like structures from human umbilical vein endothelial cells, although they had elevated levels of various angiogenesis-stimulating microRNAs—miR-92a and miR-25-3p [34].

Some members of the miR-17-92 cluster are able to enhance pro-angiogenic effects by targeting thrombospondin 1, VEGFA, and TIMP. Further, miR-20a, as a member of the miR-17-92 cluster, was shown to be able to induce increased vascular mesh and glomeruloid microvascular proliferation in BC, probably relating to overexpressed VEGFA [97]. VEGF has been shown to be implicated in regulating various microRNAs, e.g., c-Myc oncogenic miR-17-92 cluster, and it is also capable of rescuing the promoted thrombospondin-1 level, loss of endothelial cell proliferation, and development of morphological properties associated with the loss of Dicer. It is noteworthy that the pleiotropic role of miR-17-92 can be considered for breast carcinoma, which can affect angiogenesis and/or the metastatic phenotype [97] depending on the regulation of both ER pathways and tumor suppressors, as well as extracellular matrix changes [98].

An anti-ancogenic effect of some exosomal microRNAs is being widely studied as microRNAs carried by exosomes are supposed to be relevant for a targeted therapy in the future. For example, exosomes from BC cells with a lower level of Ca^2+^ were shown to contain more miR-145, targeting to IRS 1 to demonstrate an anti-angiogenic effect. Therefore, the reduction in the Ca^2+^ level in cancer cells is possibly able to contribute to antiangiogenic tumor therapy [99].

### 4.6. Immunosuppression

Immune escape of BC cells is important in the pathogenesis of BC.

Stress of the endoplasmic reticulum can be caused by a violation of protein homeostasis. MicroRNA-mediated mRNA translation inhibition has been extensively studied in the regulation of endoplasmic reticulum stress in various types of cancer. In particular, in BC, exosomal miR-27a-3p increased PD-L1 expression through the *MAGI2/PTEN/PI3K* axis, thereby contributing to immune response evasion [100]. Moreover, exosomes derived from cancer-associated fibroblasts were found to carry miR-92, which also upregulated PD-L1 expression in BC cells [101].

Tumor exosomes are able to interact with macrophages, changing their phenotype [102]. In particular, it was shown that exosomes secreted by BC cells carry glycoprotein 130 (gp130), which, together with miR-301a-3p, causes *STAT3* signaling pathway activation, resulting in an increase in reactive oxygen species concentration in macrophages [98,103]. Moreover, after interaction with tumor exosomes, the secretion of interleukin 6, TNFα, and CCL2 in macrophages increases. It was also shown that, upon inhibition of gp130, macrophages differentiate via its normal pathway. Macrophage uptake of tumor exosomes results in a loss of HLA-DR antigen and increased CD14 expression, i.e., change in the phenotype to an immunosuppressive one [104].

Besides macrophages, tumor exosomes can induce apoptosis among activated T-cells carrying the CD8 receptor [105]. This effect may be related to the expression of MHC class I on the exosomal membrane surface, which triggers T-lymphocyte apoptosis via the FasL/Fas signaling pathway [106]. For exosomes secreted by tumors, the ability to induce proliferation of suppressor cells of myeloid origin was also demonstrated, increasing its immunosuppressive abilities due to production of a variety of factors causing T-cell apoptosis [107]. Protein programmed death factor ligand 1 (PD-L1) plays an important role in suppressing the immune response: it binds to the receptor on the surface of T-cells PD-1, resulting in a suppression of proliferation and cytokine release. PD-L1 has been detected in exosomes, in co-detection with Galectin 9, which, by interacting with the Tim-3 protein, which can also be transported by exosomes, also suppresses T-cell proliferation [108].

Monoclonal antibodies to the HER2 receptor are used for first-line therapy of patients suffering from HER2/neu-positive subtypes of BC [109] However, about a year after the end of treatment, most patients become immune to the monoclonal antibody drug [110]. It was shown in vitro on cell lines SK-BR-3 and BT-474 that exosomes overexpress HER2 and are able to bind selectively to monoclonal antibodies [111]. Similar results were obtained on exosomes obtained from the blood of treated patients [110]. Probably, exosomes of HER2-positive BC subtypes competitively inhibit monoclonal antibodies, reducing their therapeutic effect. Additionally, resistance to anti-HER2 drugs has been shown to be associated with increased levels of transforming growth factor beta-1 (TGF-β1) and PD-L1 [110].

## 5. Exosomal Cargo as Source of Diagnostic Markers for Liquid Biopsy of BC

Exosomes contain a tissue-type signature wherein a rich cargo of proteins and RNA are selectively packaged. In addition, as exosomes are membranous structures, the luminal contents are protected from degradation by extracellular proteases and are highly stable in storage conditions. Since the concentration of these small vesicles in biological fluids is much higher than tumor cells (~10^8^ versus 10), exosomes are promising sources of biomarkers for the development of liquid biopsy of malignant neoplasms, including BC [112].

According to a number of studies, tumor exosomes are characterized by the increased concentration of Hsp70 protein. Fibronectin, Del-1, 20S proteasome, and, alternatively, spliced survivin, are also found in blood exosomes of BC patients [113]. In addition, a decreased representation of CD82 on the blood exosome surface of BC patients at all stages and subtypes as compared to healthy women was revealed. Moreover, the low representation of this tetraspanin in exosomes correlates with high metastatic potential of the tumor [114].

Despite the high heterogeneity of exosome proteins in both normal and pathological conditions, it is possible to identify proteins characteristic of only one of the groups.

**Figure 4:**
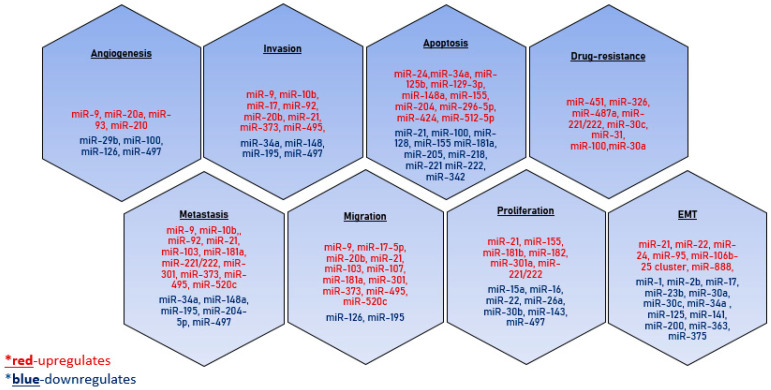
MicroRNAs involved in tumor dissemination. The microRNAs that have a stimulating effect on the development of the process are indicated in red, and the inhibitory effect is indicated in blue.

The real number of microRNAs involved in the carcinogenesis mechanisms is yet to be determined. However, it is clear that the expression profile of tissue-specific exosomal microRNAs can be successfully used to design the approaches for the early diagnosis of a tumor (Figure 4).

It should be noted that comparative analysis of the levels of tumor-specific free-circulating and exosomal microRNA revealed an increased relative level of miR-101, miR-372, and miR-373 in exosomes compared to blood serum of BC patients [115].

To date, exosomal microRNAs have been established that are significant for the diagnosis of BC, including for the differentiation of tumor subtypes (Table 2).

A serious limitation of the use of the level of exosomal microRNA in practical medicine so far is the low diagnostic significance of the proposed tests. For example, the miR-373 level in exosomes makes it possible to differentiate triple-negative BC with a sensitivity of 74.2% and a specificity of 43.6%. However, since a wide range of exosomal miR-373 levels was detected both in ER/PR-negative cases and in receptor-positive BC patients, to increase the stability of the diagnostic system, the authors proposed an expanded panel of exosomal miR-10b, -17, -34a, -93, 155, and -373 microRNAs [126].

Simultaneous analysis of different exosomal biomarkers (metabolomics markers, protein, and nucleic acids (microRNA, lncRNA, DNA, etc.) markers) can provide other levels of information about the prognosis and stage of the disease, response to therapy, and detection of minimal residual disease, increasing the overall sensitivity and specificity of the approach. However, so far, attempts to develop such approaches are known only on the basis of circulating DNA. For example, in 2018, a CancerSEEK multi-marker approach was designed based on the circulating DNA mutation search and the analysis of proteomic biomarkers (CA-125, CA19-9, CEA, HGF, MPO, OPN, PRL, TIMP-1), which makes it possible to identify eight types of cancer (ovarian, liver, stomach, pancreatic, esophageal, colorectal, lung, and breast cancer) with a sensitivity of 55% and a specificity of 99% [127].

An alternative approach to improving the results of liquid biopsy may be the development of methods for enriching exosomes of tumor origin. In this case, isolating exosomes using antibodies against tumor-associated exosomal proteins will make it possible to cut off “noise” from biopolymers from the exosomes of non-transformed cells and thus increase the sensitivity of the diagnostic systems.

## Figures and Tables

**Table 1:** Most specific exosomal proteins due to ExoCarta data (as of July 2022).

No	Gene Symbol	Number of Identifications
1	CD9	98
2	PDCD6IP	96
3	HSPA8	96
4	GADPH	95
5	ACTB	93
6	ANXA2	83
7	CD63	82
8	SDCPB	78
9	ENO1	78
10	HSP90AA1	77
11	TSG101	75
12	PKM	72
13	LDHA	72
14	EEF1A1	71
15	YWHAZ	69
16	PGK1	69
17	EEF2	69
18	ALDOA	69
19	HSP90AB1	67
20	ANXA5	67

**Table 2:** Exosomal microRNA in BC diagnosis.

Exosomal MicroRNA	Application	Expression	Reference
miR-93	Ductal carcinoma in situ diagnosis	Upregulated	[116]
miR-223-3p	Distinguish invasive ductal carcinoma from ductal carcinoma in situ	Upregulated	[117]
miR-21, miR-24, miR-206, miR-106a-363 cluster, miR-423-5p, miR-1246	Distinguish BC patients from healthy females	Upregulated	[118,119,120,121,122]
miR-18a-3p, miR-101, miR-372	Distinguish BC from benign tumors	Upregulated	[123]
miR-373	Distinguish triple-negative BC patients from luminal BC patients and healthy females	Upregulated	[124]
miR-128-1, miR-128-2, miR-340-5p, miR-421	Predict recurrence	Upregulated	[95,96,97,98,99,100,101,102,103,104,105,106,107,108,109,110,111,112,113,114,115,116,117,118,119,120,121,122,123,124,125]
miR-17-5p, miR-93-5p, miR-130a-3p		Downregulated	[95]
